# Comparison of Illumina and Oxford Nanopore Technology for genome analysis of *Francisella tularensis, Bacillus anthracis*, and *Brucella suis*

**DOI:** 10.1186/s12864-023-09343-z

**Published:** 2023-05-12

**Authors:** Jörg Linde, Hanka Brangsch, Martin Hölzer, Christine Thomas, Mandy C. Elschner, Falk Melzer, Herbert Tomaso

**Affiliations:** 1 Institute of Bacterial Infections and Zoonoses, Federal Research Institute for Animal Health, Friedrich-Loeffler-Institute, Jena, Germany; 2 Genome Competence Center (MF1), Methodology and Research Infrastructure, Robert Koch Institute, Berlin, Germany; 3 RNA Bioinformatics and High-Throughput Analysis, Friedrich Schiller University Jena, 07743 Jena, Germany

**Keywords:** Illumina, Oxford nanopore technology, R10, Genome sequencing, *Bacillus anthracis*, *Brucella*, *Francisella tularensis*

## Abstract

**Background:**

Bacterial epidemiology needs to understand the spread and dissemination of strains in a One Health context. This is important for highly pathogenic bacteria such as *Bacillus anthracis*, *Brucella* species*,* and *Francisella tularensis.* Whole genome sequencing (WGS) has paved the way for genetic marker detection and high-resolution genotyping. While such tasks are established for Illumina short-read sequencing, Oxford Nanopore Technology (ONT) long-read sequencing has yet to be evaluated for such highly pathogenic bacteria with little genomic variations between strains. In this study, three independent sequencing runs were performed using Illumina, ONT flow cell version 9.4.1, and 10.4 for six strains of each of *Ba.* *anthracis*, *Br. suis* and *F. tularensis.* Data from ONT sequencing alone, Illumina sequencing alone and two hybrid assembly approaches were compared.

**Results:**

As previously shown, ONT produces ultra-long reads, while Illumina produces short reads with higher sequencing accuracy. Flow cell version 10.4 improved sequencing accuracy over version 9.4.1. The correct (sub-)species were inferred from all tested technologies, individually. Moreover, the sets of genetic markers for virulence, were almost identical for the respective species. The long reads of ONT allowed to assemble not only chromosomes of all species to near closure, but also virulence plasmids of *Ba. anthracis*. Assemblies based on nanopore data alone, Illumina data alone, and both hybrid assemblies correctly detected canonical (sub-)clades for *Ba. anthracis* and *F. tularensis* as well as multilocus sequence types for *Br. suis*.

For *F. tularensis,* high-resolution genotyping using core-genome MLST (cgMLST) and core-genome Single-Nucleotide-Polymorphism (cgSNP) typing produced highly comparable results between data from Illumina and both ONT flow cell versions. For *Ba. anthracis,* only data from flow cell version 10.4 produced similar results to Illumina for both high-resolution typing methods. However, for *Br. suis,* high-resolution genotyping yielded larger differences comparing Illumina data to data from both ONT flow cell versions.

**Conclusions:**

In summary, combining data from ONT and Illumina for high-resolution genotyping might be feasible for *F. tularensis* and *Ba. anthracis,* but not yet for *Br. suis.* The ongoing improvement of nanopore technology and subsequent data analysis may facilitate high-resolution genotyping for all bacteria with highly stable genomes in future.

**Supplementary Information:**

The online version contains supplementary material available at 10.1186/s12864-023-09343-z.

## Background

Zoonotic bacterial pathogens are a major risk for wild animals, livestock, economy and humans worldwide [1]. Therefore, bacterial microbiologists must be able to diagnose not only genus and species, but also to distinguish bacterial organisms at the strain level, to understand their spread and dissemination in a One Health context [2]. In this regard, it is especially important to describe and understand outbreaks as well as to study routes and close sources of infections. Moreover, bacterial microbiology needs to describe the phenotype of the pathogens including potential virulence factors, resistance against antimicrobial and disinfection agents as well as their potential for horizontal gene transfer to other pathogens, e.g. via plasmids [3].

The described tasks are typical for national and international reference laboratories, which also develop and apply standards according to International Organization for Standardization (ISO) norms [4] and are especially important for monitoring the prevalence of highly pathogenic bacteria. Species of the genera *Bacillus*, *Brucella,* and *Francisella* are examples of highly pathogenic bacteria that are considered as biological agents making knowledge about their dissemination extremely important [5–7]. *Bacillus anthracis* is a Gram-positive, rod-shaped, spore-forming bacterium causing primarily cutaneous, gastrointestinal, and inhalational infections known as anthrax [8]. Major virulence factors of *Ba. anthracis* are located on two toxin-carrying plasmids (pX01 and pX02). Bacterial strains produce spores that can resist over very long periods in soil and might be inhaled by animals or humans. Other infection routes include direct contact with infected animals as well as contaminated feed or food. *Francisella tularensis* is the causative agent of tularemia, a disease which occurs in ulceroglandular, oculoglandular, oropharyngeal, or pneumonic forms [9]. In Germany, only the subspecies *holarctica* [10] occurs in natural foci and humans usually acquire the disease through contact with infected hares, but also other animals or vectors. Species of the genus *Brucella* are Gram-negative, intracellular pathogens [11]. Different species are adapted, but not restricted, to typical animal hosts such as sheep and goats (*Br. melitensis*), bovines (*Br. abortus*), pigs (*Br. suis*), and others [12]. Brucellosis is common in many countries, where it affects livestock and causes high economic losses. The bacteria are highly contagious. In humans, *Brucella* may cause severe acute febrile illness that might become a chronic disease affecting a variety of different organs [13].

The genomes of all three species used in this manuscript are considered to be stable, i.e. there is only little genetic variance between strains, also when strains with larger geographic distance are compared [10, 14–16].

Genome sequencing has been used to identify bacterial pathogens and to type bacterial strains. The advent of Next Generation Sequencing (NGS) in the 2000s [17] allowed for whole genome sequencing (WGS) of bacterial genomes. Databases and tools have been developed to detect genetic markers for virulence [18], resistance to antimicrobial and disinfection agents [19, 20], and mobile genetic elements, such as plasmids [21]. Based on WGS data, researchers can reproduce (and partly replace) commonly used standard typing approaches, such as canonical Single-Nucleotide-Polymorphism (canSNP) typing [22], classical Multilocus Sequence Typing (MLST) using 7–9 genes [23], and Multi Locus Variable copy Numbers of Tandem Repeats (VNTR) Analysis (MLVA) [24]. Since information on (almost) the entire genome is available, WGS enables high-resolution genotyping using large amounts of genomic features. Two major WGS-based high-resolution typing methods have been developed and applied: Core-genome Multilocus Sequence Typing (cgMLST), and typing based on Single-Nucleotide-Polymorphism (SNPs) [10, 14, 25].

Due to the short nature of DNA fragments („reads”) sequenced by Illumina devices, the task to assemble these short reads into complete genome sequences is challenging. In most cases, short reads alone fail to assemble complete, contiguous chromosomes and to assemble plasmids to closure [26]. Within the 2010s, a new generation of sequencing technologies was established focusing on the production of long sequencing reads [17]. ONT sequencing pulls DNA molecules through immobilized nanopores and determines the bases from the distortions in the electric current, measured as a so-called “squiggle” signal [26]. The advantage of this technology is the production of (ultra-)long reads. In fact, read lengths of up to 1 Mbp have been reported using optimized sample preparation and wet lab procedures [27]. These long reads simplify the assembly process and thus allow the reconstruction of complete and closed bacterial genomes [28]. Indeed, ONT data has recently been utilized for bacterial genotyping, both in combination with Illumina data [24, 29–32], but also without Illumina data [33, 34].

While read length, sequence throughput, and per-base sequencing accuracy have been constantly improved for ONT [26, 35], the accuracy is still lower compared to Illumina sequencing and there are systematic errors [36]. This calls for a systematic validation of ONT data to analyse bacterial outbreaks and perform genotyping which recently has been done for *Campylobacter jejuni* [37] and *Bordetella pertussis* [38].

While single strains have been sequenced using nanopore technology for *F. tularensis* [39] and hybrid assemblies were used to support *Ba. anthracis* genotyping [24], to the authors’ knowledge no systematic comparison of Illumina and ONT sequencing for *Ba. anthracis*, *Br. suis,* and *F. tularensis* has been performed yet. For this study, six DNA samples for the three species were sequenced in three runs using Illumina and ONT with flow cell version 9.4.1 and 10.4. ONT data alone, Illumina data alone, and two approaches for hybrid assembly were tested for raw data and assembly quality. Moreover, the performance for detection of virulence factors and plasmids was analysed, and commonly used standard typing approaches (MLST, canSNP, MLVA) were tested. Finally, the performance of the technologies for high-resolution genotyping (cgMLST, SNP) was evaluated.

## Results

### Quality of raw and assembled data

**Fig. 1:**
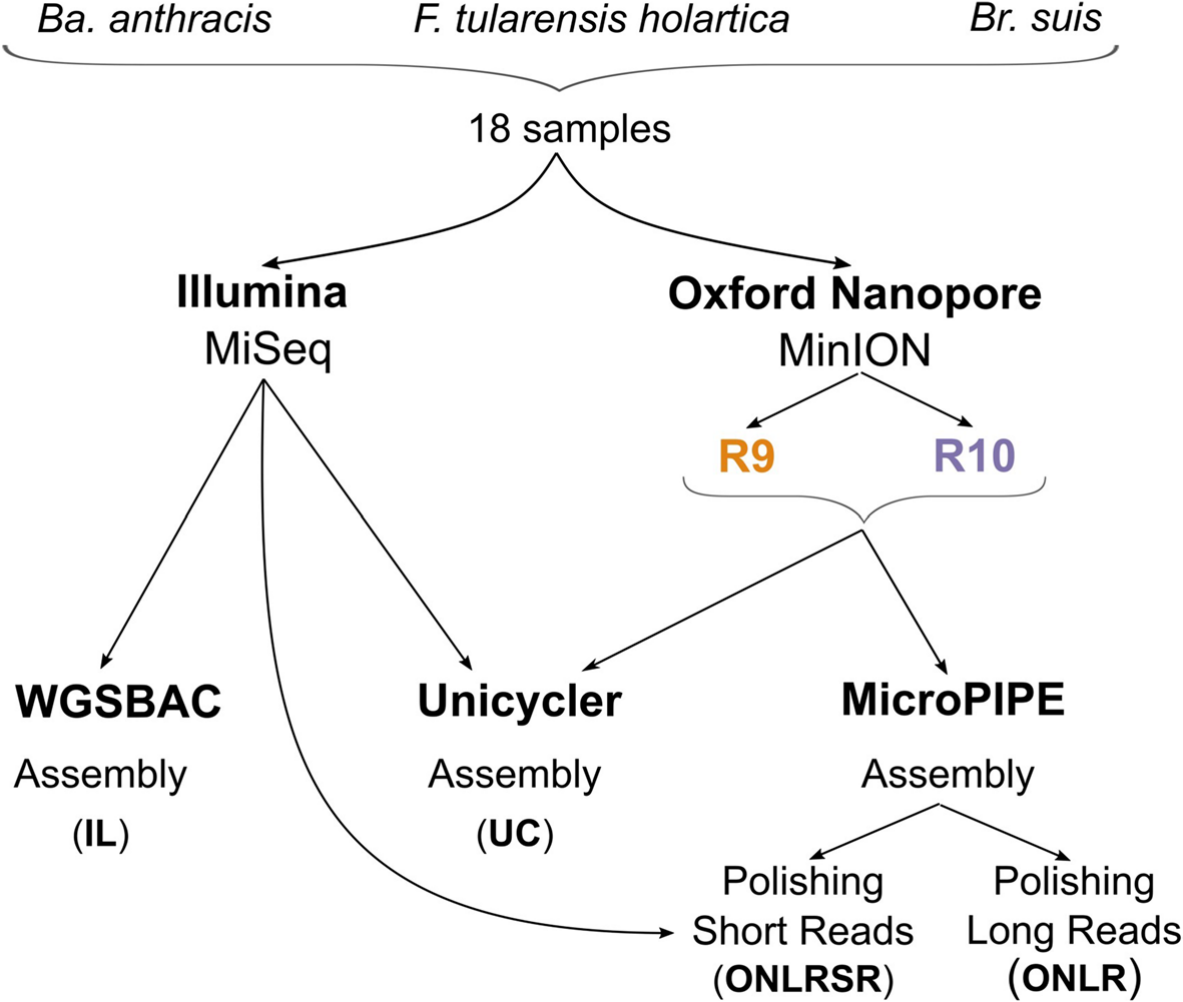
Overview of the applied workflow to compare sequencing with Illumina MiSeq to ONT MinION. DNA was extracted for six samples, respectively. Three sequencing runs for each DNA sample were performed using Illumina MiSeq (IL) and ONT MinION with flow cell version R9.4.1 (R9ONT) and R10.4 (R10ONT). Raw nanopore assemblies were polished with long reads from ONT (R9ONLR, R10ONLR) and afterwards with Illumina short reads (R9ONLRSR, R10ONLRSR). Direct hybrid assembly with Unicycler (R9UC, R10UC) was performed

For six strains of *Ba. anthracis*, *Br. suis,* and *F. tularensis* subsp*. holartica,* DNA was extracted and ONT sequencing using flow cell version R9.4.1 (R9ONT) as well as flow cell version R10.4 (R10ONT) was performed (Fig. 1). In addition, the same DNA samples were sequenced on an Illumina MiSeq (IL). The selected strains of *Ba. anthracis* were isolated from epidemiological confirmed outbreaks in 2012 and 2014 [14], while in-depth genotyping of selected *F. tularensis* strains was previously performed [10].

Raw sequencing quality (Table S1) indicated substantially more reads for MiSeq sequencing than for ONT sequencing. On the other hand, the mean read length ranged from 200 to 250 bp for Illumina, but exceeded 3,000 bp for each sample with ONT, peaking at about 16 kbp for one *F. tularensis* sample. The genomes of all three species used in this manuscript are considered to be stable, i.e. there is only little genetic variance between strains, also when strains with larger geographic distance are compared [40]. Q30 values measure the probability of an incorrect basecall in 1 out of 1,000 bases in raw sequencing reads. Depending on the species, Illumina sequencing produced on average 70–88% of bases reaching Q30. For ONT sequencing, on average 7–49% of bases reached Q15 which is equivalent to one error in 50 bp, with the proportion of bases reaching Q15 being up to six-fold higher for R10ONT compared to R9ONT. In particular, for *Ba. anthracis* it is also noteworthy that although Illumina produced orders of magnitude more reads, the overall base pair yield was 2–6 times higher for R9ONT. For the two strains, 12RA1945 and 14RA5916, R10ONT produced even more base pairs than Illumina, although the newer flow cell technology generally produced less output than R9ONT for all three species (Table S1).

All MiSeq data was assembled using Shovill (IL), while ONT raw data were assembled with Flye followed by additional polishing (Fig. 1) with the corresponding ONT reads (R9ONLR, R10ONLR). Further, two approaches for hybrid assembly were tested, a) additional polishing of ONT-polished assemblies with Illumina short reads (R9ONLRSR, R10ONLRSR) and b) direct hybrid assembly with Unicycler (R9UC, R10UC). For *Ba. anthracis,* assemblies based on Illumina data alone comprised on average 85 contigs (Table 1), with a minimum of 56 contigs (Table S2). Assemblies based on ONT data alone (R9ONLR, R10ONLR) always yielded three contigs (Table 1) except for R10ONT sequencing of strain 14RA5916 which yielded only two contigs (Table S2). Hybrid assemblies based on Unicycler yielded three contigs for five out of six strains for both ONT flow cell versions (R9UC and R10UC), respectively (Table S2). Polishing of assemblies based on R9ONT with MiSeq short reads (R9ONLRSR) yielded three contigs, except for strain 12RA1949 with seven contigs. The same assembly strategy based on R10ONT (R10ONLRSR) resulted in two contigs for strain 14RA5916, while all other assemblies consisted of three contigs. The size of the assemblies was around 40,00 bp longer when ONT data were used compared to assemblies solely based on Illumina MiSeq data, which corresponds to around 0.6% of the reference genome. A very similar GC content was detected with all sequencing and assembly strategies. The N50 value describing the contiguity of assemblies corresponded to the size of the *Ba.* *anthracis* chromosome when ONT data was used, but was smaller with Illumina alone, reflecting the higher fragmentation of short-read-only assemblies.

**Table 1:** Average quality measures of assemblies based on data from Illumina MiSeq, ONT MinION, or both. IL = Illumina only, R9ONLR = R9ONT assemblies polished with ONT reads, R9ONLRSR = R9ONT assemblies additionally polished with Illumina short reads, R9UC = Unicycler assemblies based on R9ONT and Illumina, R10ONLR = R10ONT assemblies polished with ONT reads, R10ONLRSR = R10ONT assemblies polished additionally with Illumina short reads, R10UC = Unicycler assemblies based on R9ONT and Illumina

	**Name**	**#Baseparis**	**#Contigs**	**N50 bp**	**%GC**	**% Coverage reference genome**
*Ba. anthracis*	IL	5.454.344	85,0	213.938	35,12	99,01
	R9ONLR	5.505.633	3,0	5.229.038	35,25	99,99
	R9ONLRSR	5.506.307	3,7	5.228.860	35,25	99,98
	R9UC	5.504.342	3,3	5.227.886	35,25	99,98
	R10ONLR	5.489.536	2,8	5.228.731	35,26	99,69
	R10ONLRSR	5.489.204	2,8	5.228.416	35,26	99,69
	R10UC	5.504.352	3,5	5.202.007	35,25	99,98
	Ames Ancestor	5.227.419	3,0		35,24	
*Br. suis*	IL	3.306.237	34,0	184.611	57,23	98,82
	R9ONLR	3.328.684	2,0	1.962.140	57,21	99,34
	R9ONLRSR	3.328.513	2,0	1.962.040	57,21	99,34
	R9UC	3.329.933	4,0	1.943.040	57,21	99,29
	R10ONLR	3.328.496	2,0	1.962.244	57,21	99,33
	R10ONLRSR	3.328.496	2,0	1.962.075	57,21	99,33
	R10UC	3.329.930	3,7	1.943.035	57,2	99,29
	1330	3.315.175	2,0		57,25	
*F. tularensis*	IL	1.788.491	103,8	25.987	32,17	94,21
	R9ONLR	1.894.822	1,7	1.892.558	32,17	99,75
	R9ONLRSR	1.894.774	1,7	1.892.510	32,17	99,75
	R9UC	1.876.814	1,3	1.578.270	32,17	98,92
	R10ONLR	1.892.497	1,0	1.892.376	32,16	99,75
	R10ONLRSR	1.892.460	1,0	1.892.460	32,165	99,75
	R10UC	1.871.521	1,5	1.423.518	32,17	98,65
	OSU18	1.895.727	1,0		32,16	

For *Br. suis,* the picture was similar. While assemblies based on MiSeq data alone were more fragmented, the majority of assemblies involving ONT consisted of two contigs (Table 1, S2), except for hybrid assemblies produced by Unicycler, where for two strains more than two contigs were reconstructed.

For *F. tularensis* subsp*. holartica* assemblies using nanopore data covered about 5% more of the reference genome than assemblies using only Illumina data. All assemblies for this species involving ONT data consisted of either one or two contigs (Table S2).

### Detection of genetic markers

An important step in the analysis of genome data is identifying the genus, species, and eventually subspecies of a strain and detecting genetic markers for virulence and plasmids. To this end, the average nucleotide identity (ANI) with respect to the corresponding reference genome was determined for all assemblies (Table S2). All ANI values were larger than 98% which is commonly used to define species of the considered genera [41], i.e. all sequencing and assembly strategies were able to correctly identify the species. Next, in silico PCR was performed to detect (sub-)species-specific marker genes (Table S2). All sequencing and assembly strategies detected the chromosomal marker PL3 and the plasmid marker pX01 for *Ba. anthracis*. The marker pX02 for the second *Ba.* *anthracis* specific plasmid was always detected, except for the Illumina based assembly of strain 12RA1945 as well as the ONT-based assembly (R10ONLR) and the hybrid assembly (R10ONLRSR) of strain 14RA5916 which consisted of only two contigs. With the second hybrid assembly approach based on Unicycler pX02 was detected for strain 14RA5916 in the assemblies of both ONT flow cell versions. The chromosomal marker for *Br. suis* was always detected. The marker RD-1 for *F. tularensis* always showed an amplicon-size of 924 bp characteristic for subspecies *holartica*.

Genome-based prediction of plasmids was tested for *Ba. anthracis* (Table S3) using the tool Platon, which classifies contigs as plasmid-borne or chromosomal. When using nanopore data alone or in combination with MiSeq data, mostly two contigs were predicted to be plasmid-borne. Exceptions are three contigs predicted to be plasmid-borne for strains 12RA1945 and 12RA1949 assembled using R9ONT data together with Illumina data with the tool Unicycler (R9UC). For strain 14RA5916 only one plasmid-borne contig was detected in the ONT-based assembly (R10ONLR) and the hybrid assembly (R10ONLRSR). Due to less contiguous assemblies, the plasmids were separated into 5–26 different plasmid-borne contigs (average 13) in assemblies based on MiSeq data only.

13 genetic markers for *Ba. anthracis* virulence factors were detected in all samples independent of the sequencing technology and assembly strategy, including the markers *capA*, *capB*, *capC*, and *capE* which are necessary for polyglutamate synthesis (Table S3). The only exceptions are the ONT-based assembly (R9ONLR) of strain 12RA1944 missing *pagA* and the ONT-based assemblies (R10ONLR, R10ONLRSR) of strain 14RA5916 missing *capA*, *capB*, *capC*, and *capE*, as the plasmid pX02 was not correctly assembled. Regarding *Br. suis*, for all samples, all sequencing technologies, and all assembly approaches, the same 43 virulence factors were detected.

### General typing approaches

Genotyping employing methods of the pre-WGS era was performed based on genome assemblies. CanSNP typing is based on predefined decision trees. Based on specific nucleotides at specific positions, strains are assigned to major canSNP clades and subclades. For *F. tularensis* subsp. *holartica,* all sequencing technologies and all assembly approaches detected the same major clades for the respective strains (Table S4). Moreover, the same subclades were detected for each strain, independent of the sequencing technologies and assembly strategies. All predicted major- and subclades were in accordance with previous analyses [10]. For *Ba. anthracis,* canSNP clade A.Br.002 was assigned to all strains, independent of the sequencing technology and assembly approach.

Classical MLST based on nine loci was tested with genome assemblies of *Br. suis* (Table S4). Sequence Type (ST) 16 was detected for all strains independent of the applied sequencing technology, flow cell version, and assembly approach, with two exceptions: 1) For strain 08RB3701 the assembly based on R9ONT sequencing alone (R9ONLR) did not yield any ST due to an unknown allele for the locus *trpE*. 2) The assembly based on data from a R10ONT flow cell (R0ONLR) of strain 08RB3277 detected a similar, but not identical allele for locus *dnaK*. In both cases, assemblies based on Illumina-only and the two hybrid assembly strategies predicted the correct allele.

MLVA is a molecular typing method to subtype bacterial strains based on variable number of tandem repeats (VNTRs). In silico MLVA based on assembled genomes was performed for *Ba. anthracis* and *Br. suis.* MLVA for *Ba. anthracis* is based on 32 VNTR loci (Table S5). In most cases, a specific value for each VNTR locus was detected, while in some cases the analysis resulted in no value (NA = not available). For Illumina sequencing, the average percentage of VNTRs with no values was 9%. Assemblies based on nanopore sequencing data (alone or in combination with Illumina) yielded no missing values, except for strain 14RA5916 missing three loci for assemblies based on R10ONT. For comparison, available profiles from the “Bacillus anthracis v4_1 MLVAbank” for strains 12RA1944 and 14RA5914 were used. While no value was available for the VNTR locus Bams07 in the MLVbank, the remaining experimental data was compared to in silico MLVA. On average, 83% of VNTR loci determined by Illumina sequencing were in accordance to data from MLVbank, while about 88% of loci corresponded to MLVbank considering nanopore sequencing data alone (R9ONLR, R10ONLR). Polishing ONT data with Illumina reads also yielded the largest average accordance (90%), while hybrid assembly with Unicycler reached lower accordance. For *Br. suis,* a 16-loci MLVA scheme exists. When using Illumina data alone, on average 5% of VNTR loci were not detected (Table S5). For R10ONT flow cells, an average of 3% of missing data remained, which dropped to 1% for hybrid assemblies using Unicycler.

### High-resolution genotyping

The era of whole genome sequencing facilitates using a large number of genomic features to discriminate bacteria enabling high-resolution genotyping. To this end, ONT and Illumina sequencing were compared using both established high-resolution typing methods: cgMLST and cgSNP-typing.

**Fig. 2:**
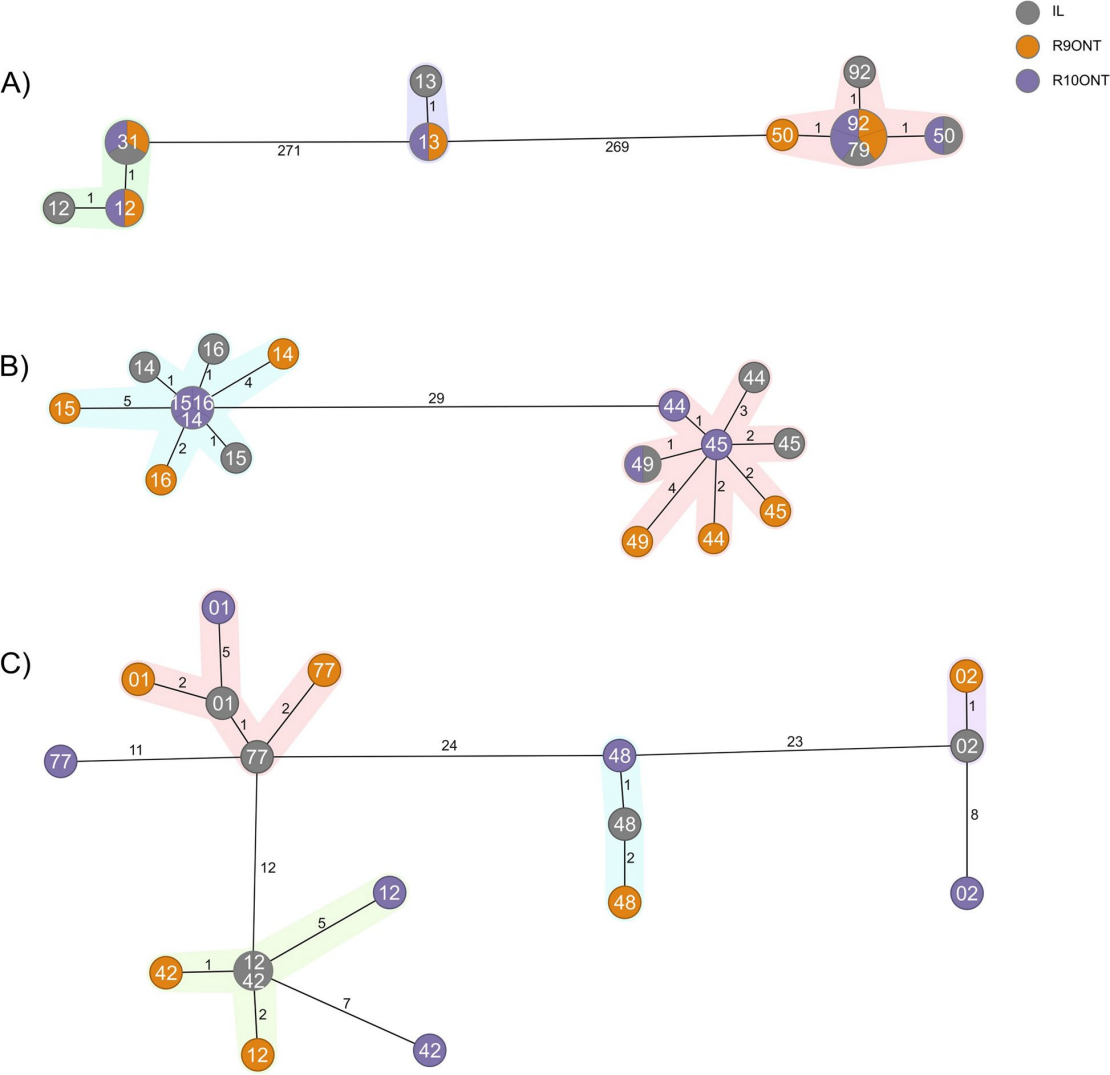
Minimum Spanning Trees visualizing allelic differences for **A** *F*. *tularensis* subsp. *holartica,* **B** *Ba. anthracis*, and **C** *Br. suis.* Nodes represent genome assemblies from Illumina, R9ONT, and R10ONT. Numbers on edges denote allelic differences. Clusters were defined with a maximum of five allelic differences and are highlighted in color. For better visibility, only last two digits of strain names are shown: 13->08T0013, 79->09T0179, 92-> 10T0192, 50 -> 12T0050, 12-> 15T0012, 31->15T0031, 02->08RB2802, 77->08RB3277, 48->08RB3448, 01->08RB3701, 12->14RB8412, 42->15RB2242,44->12RA1944, 45->12RA1945, 49->12RA1949,14-> 14RA5914, 15-> 14RA5915, 16-> 14RA5916

The *F. tularensis* cgMLST scheme contains 1,147 pre-defined cgMLST targets [42]. The applied cgMLST software defines a target (locus) as “Good target” [43] if it fulfills specific quality criteria (same length as reference genes ± 3 triplets, no ambiguities, no frame shifts). All assemblies based on Illumina data (alone or in hybrid with ONT) showed at least 98% Good Targets (Table S2). Assemblies based on MinION alone yielded on average 97% Good Targets for R9ONT sequencing, which increased to 98% for R10ONT. The Minimum Spanning Tree (MST) based on cgMLST for *F. tularensis* subsp*. holartica* clearly indicates three clusters (Fig. 2A) as previously shown for these strains [10]. CgMLST results of the different assembly types of the same strain are highly similar, independent of the applied sequencing technology, flow cell version, or assembly approach. In fact, there is no different allele for strains 15T0031 and 09T0179, no matter if they were sequenced with MiSeq, R9ONT, R10ONT or hybrid assemblies were used (Table S6). The largest distance between MiSeq sequencing (IL) and R9ONT was two alleles, which decreased to one allele comparing Illumina to R10ONT assemblies. Comparing both ONT flow cells, no differences were detected for five strains, while two alleles differed for strain 12T0050. No differences between both tested hybrid assembly strategies were observed.

The *Ba. anthracis* cgMLST comprises 3,803 pre-defined cgMLST targets [14]. Assemblies using Illumina alone or together with nanopore data yielded at least 99% Good Targets (Table S2). With at least 98%, assemblies based on MinION alone (R9ONLR, R10ONLR) reached similar quality. Again, both applied sequencing technologies were generally able to discriminate two outbreaks (Fig. 2), which were previously described [14]. The differences between R9ONT and Illumina ranged from 1 to 6 alleles, while for R10ONT smaller differences were detected to R9ONT ranging from 1–4 alleles. Again, little to no differences were detected comparing both hybrid assembly strategies (Table S6), except for strain 12RA1949, where both hybrid assembly strategies differed in four alleles to R9ONT.

Of the 1,764 cgMLST targets included in the scheme for *Brucella* spp. [25], assemblies based on Illumina (alone or combined with ONT) yielded at least 99% Good Targets while assemblies using ONT alone yielded a minimum of 93% (Table S2). In general, all sequencing technologies grouped the strains into similar clusters (Fig. 2). However, compared to *F. tularensis* and *Ba. anthracis*, for *Br. suis,* the number of differing alleles between Illumina and nanopore data was larger. There was at least one allele difference and the largest difference was eleven alleles comparing Illumina sequencing of strain 08RB3277 to R10ONT sequencing. As for the other two species under consideration, there were only subtle differences comparing both hybrid assembly strategies.

**Fig. 3:**
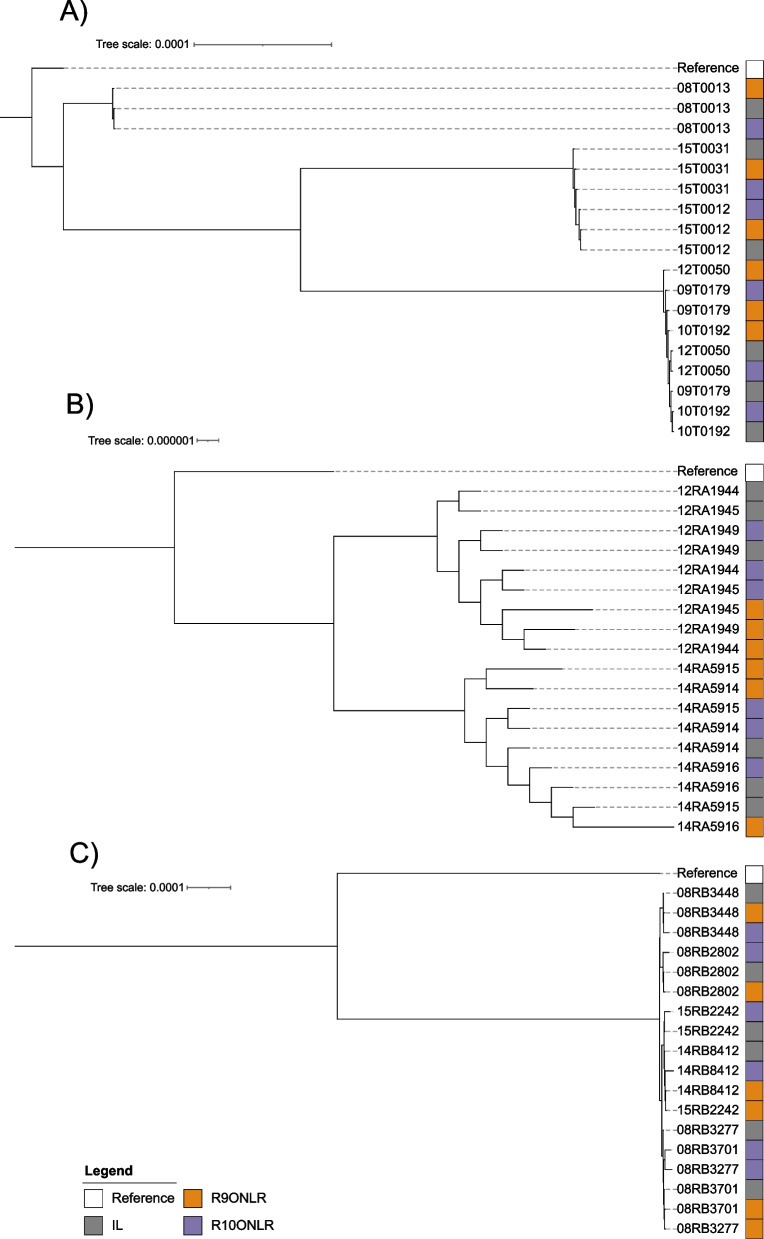
Phylogenetic trees based on core-genome SNP calling for **A**) *F*. *tularensis* subsp. *holartica,* **B**) *Ba. anthracis*, and **C**) *Br. suis.* Raw sequencing data was used for Illumina (IL) and was compared to data from long-read polished assemblies for R9ONT and R10ONT

For SNP typing, Illumina raw reads and ONT-based assemblies were used to identify SNPs compared to the reference genomes of the corresponding species followed by the detection of core-genome SNPs (cgSNPs). For *F. tularensis* subsp*. holartica,* SNP typing resulted in the same phylogeny independent of the sequencing method and flow cell version (Fig. 3). Here, all technologies grouped the samples according to established clusters [10]. In detail, there were not more than three cgSNP different between Illumina sequencing and R9ONT sequencing data, regarding strain 12T0050 (Table S6). For five strains cgSNP-typing revealed identical results comparing R10ONT with Illumina, while one cgSNP difference was detected for strain 15T0031.

The inferred phylogeny groups the six *Ba. anthracis* strains into two clades corresponding to the 2012 and 2014 outbreaks [14], independent of the sequencing technology or flow cell version (Fig. 3). There were 5–19 cgSNPs pairwise differences comparing R9ONT with Illumina sequencing. Regarding R10ONT, for five strains, no cgSNP differed in comparison to Illumina, while for strain 12RA1949 one cgSNP differed.

For *Br. suis*, SNP typing revealed similar phylogenetic topologies comparing both technologies and flow cell versions (Fig. 3). However, the number of different cgSNPs comparing the technologies was larger than for *F. tularensis* and *Ba. anthracis.* In detail, 5–15 cgSNPs differed comparing R9ONT with Illumina, while for R10ONT differences of 15–68 cgSNPS were detected.

## Discussion

This study systematically compared sequencing data generated by ONT (MinION) with Illumina (MiSeq) sequencing data for *Ba. anthracis, Br. suis* and *F. tularensis.* The three species are dangerous for livestock, the economy and humans. While other valuable studies focused on quality control and sequencing bias [36] or analysed species with rather variable genomes [37], this study analysed species with stable genomes [16, 44] and focused on tasks important for (inter-)national reference laboratories, such as the detection of genetic markers and (high-resolution) genotyping.

The raw sequencing data displayed typical features of the used technologies, where Illumina produces many short reads with high per-base quality, while ONT produces long reads with lower per-base quality. The ongoing development of ONT has been improving basecalling accuracy [35], which was demonstrated in this study by up to six-fold higher proportions of bases reaching Q15 when comparing R10ONT to R9ONT. Both sequencing technologies can be used to determine the average GC content of the considered species. As previously shown[28], ONT data is sufficient to assemble closed chromosomes and plasmids completely. This was also shown within this study. As example, it was possible to assemble two contigs for *Br. suis* corresponding to both chromosomes in all samples using data from either ONT sequencing flow cell version. Compared to the respective reference strains, ONT data covered larger parts of the reference genomes than Illumina. However, the parts of the reference genomes which were covered by ONT, but not Illumina, were negligible for *Ba. anthracis* and *Br. suis* (around 0.5%, respectively) and 5% for *F. tularensis*. As previously shown [28, 36], both technologies are able to correctly identify the species. This study supports these finding for all three pathogenic bacteria, as no significant difference in average nucleotide identity (ANI) values compared to the respective reference genomes was identified comparing ONT, Illumina, and hybrid approaches. Moreover, the species-specific chromosomal PCR markers were detected based on all assemblies.

Testing the sufficiency of ONT and Illumina data for detection and assembly of plasmids was only possible with *Ba. anthracis*, as neither *Br. suis* nor *F. tularensis* usually carry plasmids. Except for one strain, it was always possible to detect the presence of plasmids when using Illumina sequencing data alone. However, without *a priori* knowledge, the number and type of plasmids would be difficult to determine as they were dissected into many different plasmid-borne contigs in the assemblies solely based on Illumina data. In assemblies based on ONT data (alone or in hybrid with Illumina), predominantly two contigs were identified as plasmids corresponding to *Ba. anthracis* pX01 and pX02 plasmids. The PCR markers of both plasmids were found with small exceptions using data from Illumina, ONT, or their combinations. The ability of ONT to assemble closed plasmids has been demonstrated for other species in different studies [28, 30, 32] is a large advantage over Illumina data. A recent study has used the potential of ONT data to assemble plasmids and together with highly accurate Illumina sequencing data supported the identification of routes of infections in a hospital outbreak with clonal spread and cross-species exchange of plasmids [30].

Similar sets of genetic markers for virulence were found, regardless of the used sequencing technology, flow cell version, or assembly approach. This is in line with other studies, focusing on genetic markers for resistance against antimicrobial agents [28, 30, 32]. Whether ONT data alone is sufficient to also detect mutations causing resistance against antimicrobial agents [45], needs to be further elucidated.

Genotyping methods of the pre-WGS era, such as classical MLST or canSNP typing generally have a lower discriminatory power, but are still regarded when using WGS-data to i) generally classify the strains and ii) perform backward comparisons to strain collections which were not whole genome sequenced. Here, MLST typing for *Br. suis* was tested and both sequencing technologies, both flow cell versions, and both hybrid assembly approaches detected ST 16 with only two exceptions. Both exceptions were found in assemblies based on ONT alone and detected one locus, respectively, with a similar but not identical allele to an existing allele in the database. This issue might be induced by problems within the ONT data assembly process, since the integration of Illumina data during the polishing step resulted in correct. As ST 16 is the predominant ST in Germany, the detection of other STs remains untested, while the application of ONT sequencing for MLST was successfully applied for other species [28, 32].

To the authors’ best knowledge, this is the first study testing canSNP typing based on ONT data. For assemblies based on ONT data (both flow cell versions), Illumina data, and for both tested hybrid-assembly approaches, the canSNP (sub)clades of the respective strains were identical. While for *F. tularensis* subsp*. holartica* a diverse set of strains with differing canonical subtypes was tested, all *Ba. anthracis* strains belonged to clade A.Br.002, which is dominant in Germany [14]. Generally, canSNP typing for *Ba. anthracis* has little discriminatory power as merely 13 traditional canSNP clades were defined [44].

MLVA relies on the analysis of genomic repeats (VNTRs). The utilization of WGS data for *in silico* MLVA was first performed for *Br. melitensis *[46]. A comparison of *in silico* MLVA with PCR fragment analysis indicated high accordance [47]. While Illumina short reads might sometimes not be sufficient to assemble VNTR loci within one contig, it has remained elusive whether the sequencing accuracy of ONT is sufficient for MLVA. There are two possible reasons for receiving no value (NA) for specific VNTR loci in MLVA. First, at least one or both primer target sequences cannot be detected in the genome assemblies. Second, both primer target sites can be detected, but are located on different contigs, thus, no product size can be determined. This study indicated a slightly larger fraction of missing values for assemblies based on Illumina data compared to assemblies based on ONT data. Combining data from both sequencing technologies resulted in the smallest fraction of missing values. For *Ba. anthracis* results from conventional MLVA downloaded from MLVAbank were compared to WGS-based results. For VNTR locus pX02_at, all sequencing technologies predicted value 9, while conventional MLVA found 10. As the repeat length of this locus is merely 2 bp, the differentiation between alleles might be error-prone using conventional MLVA. For locus Bams15 conventional MLVA differs from all assembly-based results. This locus was initially thought to consist of 18-mer repeats, but later turned out to be only 9-mer leading to inconsistent allele numbers [48]. For the remaining loci, this study proved higher accordance with conventional MLVA to assemblies based on ONT data alone than for assemblies based on Illumina alone. Again, the best results were achieved when combining both technologies.

Regarding genotyping and outbreak analysis, the biggest advantage of WGS-based methods over pre-genomic methods is their potential to perform genotyping at higher resolution [10, 14, 15, 23, 25, 47], which may help to distinguish strains based on one SNP or allele difference. While cgMLST based on Illumina data has been successfully applied for genotyping of the three species under consideration before [10, 14, 24], this study, for the first time, employed ONT sequencing for this task. For *F. tularensis* subsp*. holartica* this study demonstrated highly comparable cgMLST results regarding Illumina and ONT sequencing data. Moreover, only subtle allele differences between data from both tested flow cell versions were discovered and all sequencing technologies clustered strains from previous outbreaks [10]. Given this negligible differences between sequencing technologies, high-resolution genotyping for *F. tularensis* subsp*. holartica* might be possible even for datasets mixing both technologies. Also for *Ba. anthracis,* cgMLST based on Illumina and ONT was able to group strains according to the known outbreaks [14]. However, the number of allelic differences for repeated sequencing was slightly higher compared to *F. tularensis.* In addition, there were slight, but important differences comparing both flow cell versions. Compared to Illumina, some strains showed more than five alleles difference for R9ONT, while for R10ONT all differences were smaller than this cut-off. Though defining specific cut-offs for genotyping is an ongoing process, clustering based on five alleles has been shown to detect outbreak clusters for *Ba. anthracis* [14]. Regarding *Br. suis*, cgMLST grouped the strains into four different clusters. However, the number of allelic differences between sequencing technologies, but also between flow cell versions was increased in comparison to the other two species under consideration. Further studies are needed to analyse if cgMLST mixing Illumina data and ONT data is feasible for *Br. suis.*

Similar to cgMLST, SNP typing is a high-resolution genotyping method that has been successfully applied to all species under consideration [10, 15, 23, 24, 47]. As for cgMLST, SNP typing for *F. tularensis* subsp*. holartica* produced highly comparable results with both sequencing technologies. Moreover, the number of cgSNPs for R10ONT compared to Illumina was even smaller than it was for R9ONT. This progress in nanopore design and concomitant quality might, in the future, enable SNP typing of datasets from both technologies with identical accuracy. For *Ba. anthracis,* the phylogeny based on SNPs revealed lineages corresponding to known outbreaks [14] for both sequencing technologies and both flow cell versions. Compared to Illumina sequencing, data based on R10ONT produced less cgSNPs than data based on R9ONT. In contrast, although for *Br. suis* the general phylogeny based on ONT-only data was comparable to Illumina data, the number of cgSNPs was higher comparing both technologies and no improvement was detected using R10ONT.

One possible reason for the better agreement between the sequencing technologies for *F. tularensis* and *Ba. anthracis* compared to *Br. suis* might be the differences in GC content which exceeds 50% for *Br. suis,* but 32% and 35% for the other two species, respectively (Table 1). Lately, reads with high GC content have been suggested to be a systematic source of errors [36]. The R10ONT libraries for *Br. suis* indicate lower genome coverage than R9ONT. While further research is needed to obtain a cut off for minimal coverage, the coverage is still larger than 20 which was recently shown to be sufficient for bacterial genomics [40].

This manuscript used Snippy [49], a pipeline for bacterial haploid variant calling and core genome alignment which allows comparing SNP distances for sets of strains and building phylogenies. Although ONT-specific tools for variant calling exist, they do not perform core genome alignment [50] or have not been optimized [51, 52] or tested [53, 54] for bacteria. Therefore, we decided also to use Snippy on the ONT data but use the assembled and polished contigs as input rather than the erroneous raw long reads.

The major advantage of ONT data is the possibility to assemble complete and closed genomes, as shown in this manuscript and previously [28]. In this way, all genes, can be sequenced and arranged, but an important further advantage is the complete construction of plasmids [30]. On the other hand, nanopore technology has produced more sequencing errors than Illumina. Recent improvements in technology and bioinformatics, however, have dramatically reduced those sequencing errors [17]. Though there has been an improvement in average read accuracy, ONT data may still suffer from systematic errors [36], such as homo- and heteropolymer genomic regions, methylation, and high GC. This study compared R9ONT flow cells with R10ONT and demonstrated improved basecalling accuracy for R10 chemistry. While for *F. tularensis* and *Ba. anthracis* this improvement led to better comparability with genotyping based on Illumina reads, this was not the case for *Br. suis*. Moreover, the R10 chemistry required a significantly larger amount of input DNA at the time when writing this manuscript. R10.4 chemistry will surely be further improved, nevertheless, polishing nanopore assemblies with Illumina data might be used to achieve the highest possible quality of assemblies [55].

In this manuscript, two strategies for combining ONT and Illumina data in hybrid assemblies were compared. With MicroPIPE [56] long ONT reads are initially assembled, afterwards long reads are used for polishing and finally those assemblies are polished again with Illumina short reads, using independent tools for each step. The theoretical advantage is that the initial assembly will be constructed based on the long reads, taking advantage of their higher contiguity. On the other hand, Unicycler [57] uses long and short reads together, starting from an initial short-read-based *de Brujin* graph, which is then further refined using the long reads. However, and as previously shown, for many use cases there is little difference between both approaches regarding the quality of assemblies [56]. Although this study identified small differences between both approaches for some samples regarding the detection of genetic markers or genotyping, the overall results remain similar.

While constant improvements facilitate genomic analysis of bacterial isolates with highly stable genomes, purely using nanopore data in the future, combined with real-time basecalling and adaptive sequencing [58], might continue to revolutionize molecular diagnostics. During ONT runs sequence data is immediately available. This so-called “real-time sequencing” enables direct pathogen identification from metagenomic DNA isolated from patients, animals or the environment [59]. Real-time sequencing may also improve emergency responses by dramatically speeding up genomic characterization of bacterial pathogens of public health concern, such as *Ba. anthracis* [60]. Finally, adaptive sequencing can improve enrichment of target DNA sequencing reads from complex samples. This approach combines ONT real-time sequencing with real-time data analysis. During passaging of a DNA molecule through a nanopore, the first part of its sequence is being analysed in real time. If this sequence is not part of a predefined set of targeted DNA sequences, a software initiates the rejection of the DNA molecule by the pore. Using adaptive sequencing, enrichment of target species for more than 13-fold has been demonstrated [61, 62]. Application of adaptive sequencing to deplete host DNA reads in patient samples is likewise feasible [61]. In a pioneering manuscript, the capability of adaptive sequencing to not only enrich for species but also for specific genetic markers such as those for antimicrobial resistance genes was demonstrated [63].

## Conclusion

In summary, this study showed highly comparable results comparing ONT with Illumina, with increasing sequencing quality for ONT flow-cell version R10.4. Both sequencing technologies detected nearly identical sets of genetic markers, while ONT data allowed to assemble plasmids to near closure. Applying general typing approaches (e.g. MLST, canSNP typing) seems possible with both technologies. Combining data from ONT and Illumina for high-resolution genotyping might be feasible for *F. tularensis* and *Ba. anthracis,* but not yet for *Br. suis.* The ongoing improvement of nanopore technology and subsequent data analysis may facilitate high-resolution genotyping for all bacteria with highly stable genomes in future.

## Methods

### Culturing and DNA Extraction

Six strains of *F. tularensis* subsp. *holarctica* were cultivated on cysteine heart agar (CHA, Becton Dickinson, BD Heidelberg, Germany) at 37 °C with 5% CO_2_ for 72 h. The DNA used for whole genome sequencing was prepared using the QIAGEN Genomic-tip 20/G Kit (Qiagen GmbH, Hilden, Germany). The DNA extraction was performed according to the instructions of the manufacturer for sample preparation and the lysis protocol for bacteria using 1 ml buffer B1 with 2 µl RNase A, 45 µl proteinase K, and 20 µl lysozyme.

Cultivation of 6 strains each of *Ba. anthracis* and *Br. suis* biovar 2 was performed at 37 °C on nutrient agar (Merck, Darmstadt, Germany) for 24 h and on nutrient agar with 7.5% calf blood for 48 h, respectively. High molecular weight DNA was extracted using the NucleoBond HMW DNA kit (MACHEREY–NAGEL, Düren, Germany).

### Sequencing

In sum, 54 sequencing datasets were generated (Fig. 1). DNA from six strains of *F. tularensis* subsp. *holarctica*, *Ba. anthracis*, and *Br. suis* was extracted and sequenced in three independent runs, for each species respectively: A) The first run used ONT flow cells of version R9.4.1 (called R9ONT hereafter). Here, the Ligation Sequencing Kit SQK-LSK 109 (Oxford Nanopore Technologies Ltd, Oxford, England) in combination with the Barcoding Kit EXP-NBD 104 was used for library preparation. B) The second run utilized ONT flow cells of version R10.4 (called R10ONT). Therefore, libraries were prepared with the Ligation Sequencing Kit SQK-LSK 112.24 for R10 chemistry. All ONT libraries were run on a MinION Mk1B device for 24 h. C) Finally, one run performing 300 bp paired-end sequencing with an Illumina MiSeq (called IL) was performed, for which libraries were prepared using the Nextera XT kit (Illumina Inc., San Diego, CA, USA). All libraries were prepared according to the manufacturer’s instructions.

### Bioinformatics data analysis

Data analysis was applied to 36 sequencing datasets (based on six samples per species) generated with ONT MinION and 18 datasets generated with Illumina MiSeq. All nanopore data was processed with the pipeline MicroPIPE [56] using all tools with standard settings if not mentioned otherwise. Guppy [64] v6.0.1 was used for basecalling. The utilized model was dna_r9.4.1_450bps_sup for R9ONT and dna_r10.4_e8.1_sup.cfg for R10ONT. Guppy was also used for demultiplexing and trimming of the barcodes. Porechop [65] v0.2.3_seqan2.1.1 was used for adapter trimming and Japsa [66] v1.9-01a for quality filtering using a minimal length of 1,000 bp and a minimal quality of 10. For assembly of quality trimmed ONT data, Flye [67] v2.5 was used with asm-coverage 50. Specifically for *Ba. Anthracis,* Flye was used in plasmid mode. Raw assemblies produced by Flye were polished with the corresponding long reads using one round of Racon [68] v1.4.9 and one additional polishing round with Medaka [50] v0.10.0 to produce final polished ONT-based assemblies (called R9ONLR and R10ONLR).

Two strategies for hybrid assemblies combining ONT and Illumina data were tested. The first strategy is based on MicroPIPE [56]. After polishing assemblies from Flye with long ONT reads, NextPolish [69] v1.1.0 was used for another polishing round with MiSeq short reads (called R9ONLRSR and R10ONLRSR). The second, strategy performed direct hybrid assembly utilizing raw data from MinION and MiSeq with Unicycler [57] v0.4.8 (called R9UC and R10UC).

Raw paired-end Illumina data was analysed with the pipeline WGSBAC [10, 15, 24, 25] v2.2.0. Data quality was controlled by WGSBAC with FastQC [70] v0.11.5 and raw coverage was calculated as the number of reads multiplied with their average read length and divided by the genome size. Based on raw Illumina reads, Shovill [71] v1.0.4 performed quality trimming, adapter trimming, and assembled reads into contigs (called IL).

All assemblies (ONT, Illumina, hybrid) were further analysed with WGSBAC. This included quality control and comparison of assembly metrics QUAST [72] v5.0.2. The tool pyani [73] calculated Average Nucleotide Identity (ANI) of all assemblies compared to their corresponding reference genomes: *Ba.* *anthracis* Ames Ancestor (accession ASM844v1)*, Br. suis* 1330 (accession ASM750v1), and *F. tularensis* subsp*. holartica* OSU18 (accession ASM1140v1).

Species-specific target genes were detected with in_silico_PCR [74] v 0.5.1. This included the primers to detect *Ba. anthracis* chromosomal DNA (PL3) [75] as well as plasmids pX01 and pX02 [55], and *Br. suis* chromosomal DNA (BS1330_II0657) [76]. For *F. tularensis* the chromosomal marker RD-1 was used, and the amplicon was checked for the specific length (924 bp) of the subspecies *holartica*.

For the detection of genetic biomarkers for virulence, WGSBAC ran ABRicate [77] v0.8.10 together with the Virulence Factor Database (VFDB) [18]. Plasmid-borne contigs were identified with the tool Platon [21] v1.5.0.

The tool CanSNPer [22] v1.0.8 was used based on assemblies for pre-defined canonical Single-Nucleotide Polymorphism Typing (canSNPs) for *Ba. anthracis* and *F. tularensis*. For *Br. suis,* WGSBAC performed classical MLST (nine loci) based on assembled genomes using the software mlst [78] v2.16.1. MLVA was performed based on genome assemblies with the tool MISTReSS [79] using established MLVA-schemes for *Ba. anthracis* [24] and *Brucella* [46]*.* For comparison, available MLVA profiles from strain 14RA5914 (Dobichau) and 12RA1944 (Stendal) were downloaded from the Bacillus anthracis v4_1 MLVAbank [80].

The software Ridom Seqsphere + [43] v8.2.0 was used for cgMLST together with the specific core-genome scheme for *Ba. anthracis* [14], F. tularensis [42], and *Brucella* spp. [25], respectively. Ridom Seqsphere + was also used to construct Minimum Spanning Trees (MSTs).

Core-genome SNP (cgSNP) calling for Illumina data was performed using Snippy [49] v. 4.3.6 in standard settings. Snippy performs read mapping against the respective reference genomes (*Ba*. *anthracis* Ames Ancestor, *Br. suis* 1330, *F. tularensis* subsp*. holartica* OSU18), SNP calling, filtering, and finally, identifies cgSNPs of the provided sets of samples. For Illumina data, raw reads served as input, while for nanopore data, assembled contigs after polishing with ONT long-reads (R9ONLR, R10ONLR) were used. Snps-dists v0.63 [81] was used to calculate pairwise SNP distances based on the cgSNP alignment. Reconstruction of phylogenetic trees based on the cgSNP alignment was performed via RAxML (Randomized Accelerated Maximum Likelihood) v8 [82]. The interactive Tree of Life (iTOL) v. 4 web-tool [83] was used for visualization of the trees.

## Supplementary Information

**Additional file 1: Table S1. **Quality measures of raw sequencing data.

**Additional file 2: Table S2. **Quality measures of assembled genomes.

**Additional file 3: Table S3. **Genetic markers for virulence and plasmids.

**Additional file 4: Table S4.** Canonical SNP (canSNP) -typing, Multilocus Sequence Typing (MLST).

**Additional file 5: Table S5. **Multi Locus Variable Copy Numbers of Tandem Repeats Analysis (MLVA).

**Additional file 6: Table S6. **Pairwise distances of cgMLST and cgSNP typing.

## Data Availability

The sequencing data used in this manuscript has been deposited with the European Nucleotide Archive and is available under Bioproject PRJEB59317.
